# Spontaneous Uterine Rupture Secondary to Morbidly Adherent Placenta in an Unscarred Uterus

**DOI:** 10.7759/cureus.7281

**Published:** 2020-03-15

**Authors:** Nida Sajjad, Kumail Khandwala, Wasim A Memon, Jehanzeb Shahid, Burhan Zafar

**Affiliations:** 1 Radiology, Aga Khan University Hospital, Karachi, PAK

**Keywords:** morbidly adherent placenta, uterine rupture, placenta accreta, computed tomography

## Abstract

We report a case of spontaneous uterine rupture in a primigravida with an unscarred uterus, which was secondary to morbidly adherent placenta proven on surgery and histology. Although rare, uterine rupture should be considered as a differential diagnosis of acute abdominal pain in pregnancies, especially when associated with free fluid, even with the absence of vaginal bleeding. Abnormal placentation is associated with spontaneous antepartum uterine rupture even in early pregnancy. Most cases in the literature have advocated emergency hysterectomy to arrest life-threatening hemorrhage.

## Introduction

Uterine rupture during pregnancy is a rare and often catastrophic event, with a previously reported incidence of one in 1,536 pregnancies [1]. It is particularly rare in unscarred uteri. The overall incidence of uterine rupture in unscarred and scarred uteri is 0.7 and 5.1 per 10,000 deliveries, respectively [1,2]. Associated maternal and fetal morbidity and mortality is high. We report a case of spontaneous uterine rupture in a primigravida woman with an unscarred uterus, which was secondary to morbidly adherent placenta.

## Case presentation

**Figure 1:**
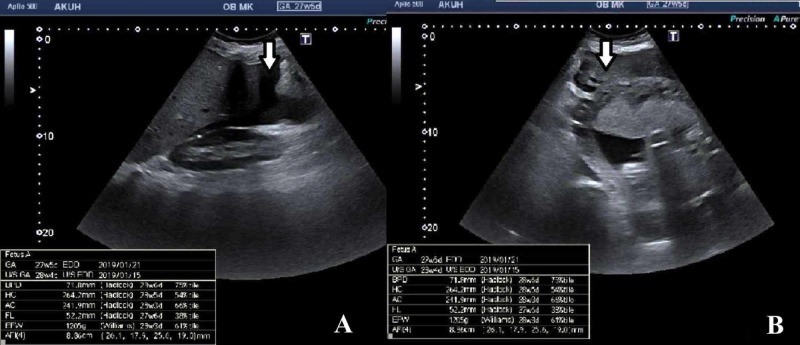
Ultrasound images. Free fluid with internal echoes (arrows) was noted adjacent to Morison's pouch (A) and uterus (B).

A 34-year-old lady, primigravida, with no known prior comorbids or history of trauma, was admitted to the emergency department of our hospital for sudden onset of acute abdominal pain. She otherwise had an uneventful antenatal course. On examination, she was tachycardic and hypotensive, and had abdominal lower abdominal tenderness with guarding. On ultrasound, a single alive fetus with fetal growth parameters corresponding to 28 weeks and four days was seen in the uterine cavity. There was free fluid with internal echoes adjacent to the uterus extending to Morison's pouch, suggestive of hemoperitoneum. Amniotic fluid index was on the lower side, measuring 8.8 cm and corresponding between 2.5th to 5th percentile for this gestational age. The placenta was anteriorly positioned in the upper uterine segment; however, the other characteristics of the placenta and distance from the cervical os could not be evaluated sonologically because the patient did not have a full urinary bladder at the time of scanning. Based on the clinical examination and hemodynamic instability of the patient, differentials of ruptured hemorrhagic ovarian cyst and acute appendicitis were also given based on the sonographic findings of free intraperitoneal fluid containing internal echoes (Figure 1). 

**Figure 2:**
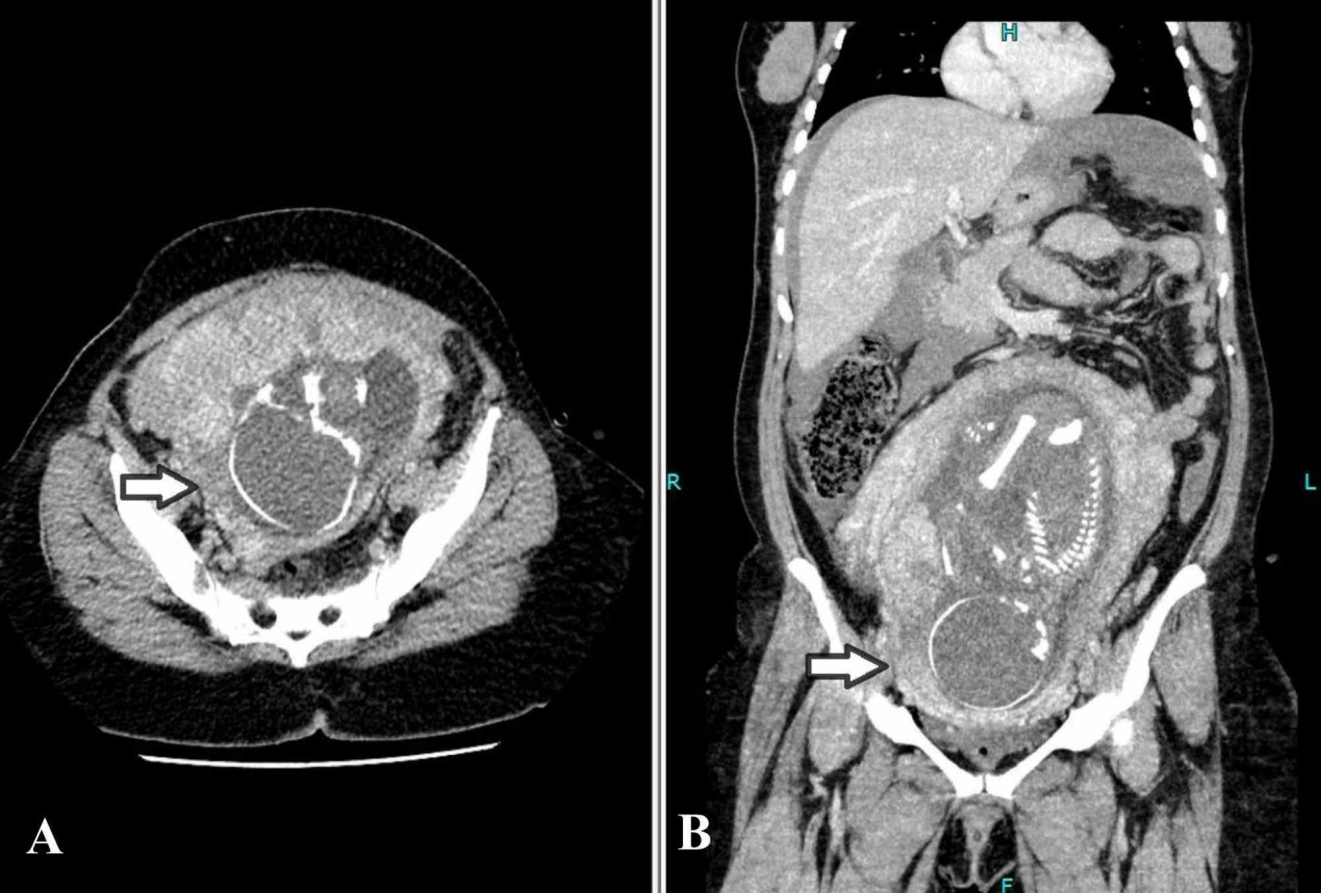
Computed tomography images: (A) axial and B) coronal sections. Gravid uterus with myometrium not clearly appreciated inferolaterally on the right side at site of placental attachment, which was raising the possibility of placenta accreta with uterine rupture (arrows).

**Figure 3:**
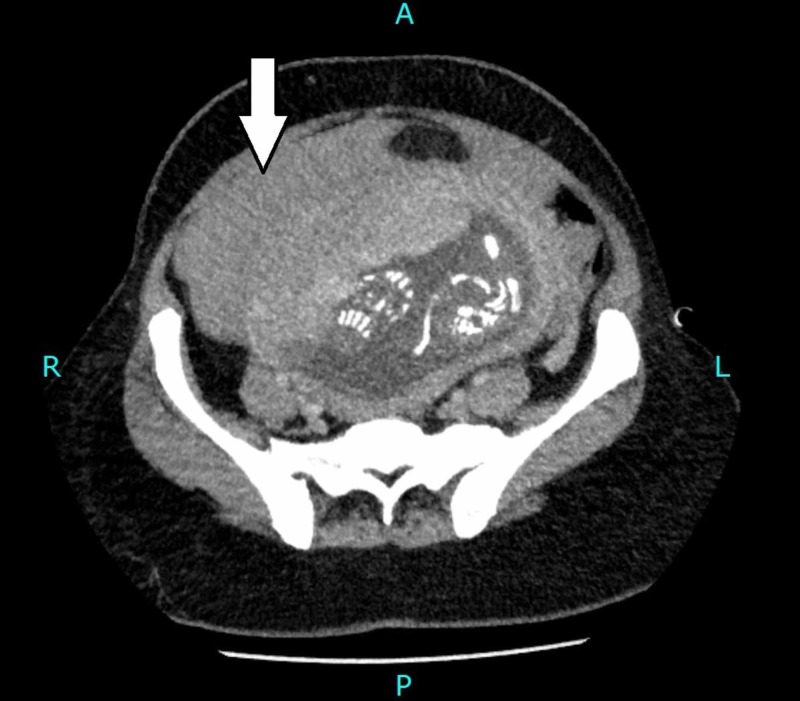
Computed tomography image (axial section). High-density free fluid with Hounsfield units of 80, adjacent to the uterus, suggestive of hemoperitoneum (arrow).

Magnetic resonance imaging (MRI) was initially considered but was not technically possible due to time constraints and unstable hemodynamics of the patient. After taking high-risk consent and explaining the fetal radiation risk to the patient's family in detail, emergency computed tomography (CT) scan was then performed which showed a gravid uterus with single intrauterine pregnancy. The placenta was noted along the anterior wall of uterus in the right lateral position. The myometrium was not clearly appreciated inferolaterally on the right side at site of placental attachment, which was raising the possibility of placenta accreta (Figure 2). There was evidence of hemoperitoneum with Hounsfield units of 80 (Figure 3). Appendix was not separately visualized due to hemoperitoneum.

The patient was rushed to the operating room. Intraoperatively, a rent was noted in the anterior inferior uterine wall consistent with uterine rupture, and a uterus-saving procedure was found to be impossible. A subtotal hysterectomy and bilateral salpingectomy remained the only surgical options. During the operation, the patient suffered a blood loss of approximately 2,500 mL. The uterine structures were sent for further microscopic evaluation. Histology showed the placental tissue was adherent to myometrium, with evidence of invasion and focal serosal distortion at the site of rupture. Sections examined from endomyometrium revealed chorionic villi. These villi were in direct contact with the underlying myometrium without intervening decidua. The findings were in keeping with morbidly adherent placenta (placenta accreta).

The fetus was born alive, and admitted to the neonatal intensive care unit for further management. The patient was discharged in a stable condition on the fourth post-operative day.

## Discussion

Uterine rupture of the primigravid unscarred uterus is an extremely rare entity. The estimated incidence of spontaneous rupture of unscarred uteri in developed countries is approximately 12 in 100,000 pregnancies [3,4].

The most common and important risk factor for uterine rupture is a scarred uterus from previous uterine surgery, most commonly cesarean section. Other causes include prolonged labor, congenital uterine anomalies, a morbidly adherent placenta, use of oxytocin and prostaglandin analogs, connective tissue disorders, and adenomyosis [4,5]. In our case, the patient may have been unwilling to disclose medical or surgical history that could have included previous uterine instrumentation such as curettage; however, she claimed this was her first pregnancy.

Other non-gynecological and non-obstetric causes of acute abdominal pain with hemoperitoneum such as perforation of bowel, acute appendicitis, acute pancreatitis, and rupture aortic aneurysm should also be considered and ruled out before making a definitive diagnosis [6]. This case report emphasizes the difficulties in early diagnosis and management in cases of ruptured uterus in unscarred uterus in non-laboring women.

Uterine rupture has been reported in primigravidas with placenta percreta in both the second and third trimesters [7]. The site of uterine rupture involves the lower uterine segment in late gestation, but the uterine fundus is commonly affected in the first trimester [6]. In our patient, the site of rupture was inferolateral where the uterine wall was invaded by the placental trophoblastic tissue, making it weak and thin. In late trimester pregnancies, focal ruptures of the uterine wall may be difficult to discern on ultrasound. In addition, a previously published case series suggest that the signs and symptoms of uterine rupture are typically non-specific, which additionally makes diagnosis difficult [4]. Theilen et al. challenged the American College of Radiology recommendation of ultrasound as the initial imaging modality for appendicitis in pregnant patients and concluded that MRI is the investigation of choice in this patient population, in settings in which MRI is readily available [8]. However, in their study, none of the 171 pregnant patients with acute abdominal pain similar to our patient had evidence of uterine rupture, which makes our case unique. Additionally, MRI was not readily available for our patient from a logistical standpoint; thus, emergency CT was performed. A similar situation has also been reported previously by Moussa et al. [9].

Emergency surgery is usually required to control hemorrhage and to save the patient's life. Use of conservative surgery has also been reported with bilateral uterine vessel ligation along with excision of the ruptured uterine segment to preserve the uterus. However, in case of excess hemorrhage not controlled by simple suturing, it is suggested that early decisions for hysterectomy should be taken to prevent morbidity and mortality. Most cases in the literature report total to subtotal hysterectomy to arrest hemorrhage [5-7].

## Conclusions

Although rare, uterine rupture should be considered as a remote differential diagnosis of abdominal pain in early trimesters, especially when associated with free fluid, even with the absence of vaginal bleeding. Abnormal placentation is associated with spontaneous antepartum uterine rupture even in early pregnancy.

## References

[1] Mourad WS, Bersano DJ, Greenspan PB, Harper DM (2015). Spontaneous rupture of unscarred uterus in a primigravida with preterm prelabour rupture of membranes. BMJ Case Rep.

[2] Kashyap P, Prasad S, Singh BC (2017). A rare case of second trimester uterine rupture in unscarred uterus. Int J Med Res Health Sci.

[3] Kaur J, Goel B, Sehgal A (2012). Rupture uterus following blunt trauma at 16 weeks gestation. Int J Reprod Contracept Obstet Gynecol.

[4] Revicky V, Muralidhar A, Mukhopadhyay S, Mahmood T (2012). A case series of uterine rupture: lessons to be learned for future clinical practice. J Obstet Gynaecol India.

[5] Uccella S, Cromi A, Bogani G, Zaffaroni E, Ghezzi F (2011). Spontaneous prelabor uterine rupture in a primigravida: a case report and review of the literature. Am J Obstet Gynecol.

[6] Ronen JA, Castaneda K, Sadre SY (2018). Early accreta and uterine rupture in the second trimester. Cureus.

[7] Siwatch S, Chopra S, Suri V, Gupta N (2013). Placenta percreta: rare presentation of haemorrhage in the second trimester. BMJ Case Rep.

[8] Theilen LH, Mellnick VM, Longman RE (2015). Utility of magnetic resonance imaging for suspected appendicitis in pregnant women. Am J Obst Gynecol.

[9] Moussa HN, Nasab SH, Blackwell SC, Sibai BM (2016). Uterine rupture in pregnancy: imaging beyond ultrasound. J Neonatal Biol.

